# Comparison of Three Urine Normalization Strategies for Mass Spectrometric Analysis in a Pediatric Asthma Model

**DOI:** 10.21627/3p72dt97

**Published:** 2026-09-15

**Authors:** Emma Finch, Maryam Alyari, Mays Al-Dulaymi, Anas El-Aneed, Darryl J. Adamko

**Affiliations:** Department of Pediatrics, College of Medicine, University of Saskatchewan, Saskatoon, Saskatchewan, Canada; Department of Pediatrics, College of Medicine, University of Saskatchewan, Saskatoon, Saskatchewan, Canada; College of Nutrition & Pharmacology, University of Saskatchewan, Saskatoon, Saskatchewan, Canada; Department of Pediatrics, College of Medicine, University of Saskatchewan, Saskatoon, Saskatchewan, Canada; College of Nutrition & Pharmacology, University of Saskatchewan, Saskatoon, Saskatchewan, Canada; Department of Pediatrics, College of Medicine, University of Saskatchewan, Saskatoon, Saskatchewan, Canada

**Keywords:** asthma, CHILD Cohort Study, creatinine normalization, creatinine-to-height normalization, LC-MS normalization, pediatric cohort, urine metabolomics

## Abstract

INTRODUCTION: Metabolomics studies the products of metabolism within a biological system to determine biomarkers for disease diagnosis. Metabolite values found in urine analysis require a method to normalize the data to account for urine concentration. Creatinine (CRN) is a commonly used method of LC-MS/MS data normalization; however, CRN is affected by muscle mass and height (HT) in children. This could be a concern when comparing children’s samples across various age groups. Referencing CRN to HT (CRN/HT) is a possible strategy to correct for such a concern. Another approach is to reference each metabolite to the total concentration of the metabolites, a strategy termed the total metabolome (TM). METHODS: We evaluated age/HT as a confounder in pediatric metabolomic data analysis by comparing CRN, CRN/HT, or TM normalization in children aged 3–13 with or without asthma (n = 152). LC-MS/MS measured 41 metabolites in urine samples, which were normalized to the three methods. These metabolites were then used to differentiate children with asthma versus healthy children. RESULTS: CRN was affected by age, unlike CRN/HT or TM. The metabolites selected to differentiate healthy and asthmatic children differed based on the normalization technique; 10 metabolites were shared across models. Diagnostic accuracy was affected by the normalization strategy. CONCLUSION: Using CRN/HT or TM limits the variation seen across ages and was better than CRN alone. The normalization strategy for hydration affects model accuracy in pediatric research.

## Non-Specialist Summary

There is a need for better diagnosis of asthma in children. Metabolomics studies the products of metabolism within a biological system to determine biomarkers for disease diagnosis. Urine contains many potential diagnostic biomarkers, but their quantity is impacted by the urine concentration of the child. To correct for this, urine data are normalized to creatinine. While studying different ages of children, we became concerned that creatinine was not adequate because it was affected by age and height. This article describes the issue and two possible solutions to address creatinine shortcomings within pediatric populations.

## 1. Introduction

Asthma is the most common chronic disease in children, occurring in 14% of children worldwide [1–3]. It is a respiratory disease characterized by narrowing of the airways and as a result leads to difficulty breathing [3, 4]. Current asthma diagnosis is largely based on assessing the patient’s history. Objective tests like spirometry, fraction exhaled nitrous oxide, or bronchoprovocation may help, but they are not used in most doctors’ offices [1–3, 5]. In addition, these tests are not applicable to children under the age of 5 years because they are unable to perform them properly [1, 3]. The diagnosis of asthma can be delayed due to the lack of objective testing. In fact, multiple asthmatic attacks may occur before a definitive diagnosis is attained as each presentation of an attack contributes to the diagnosis [1–3]. A better diagnostic test is needed.

Metabolomics studies the products of metabolism within a biological system to determine biomarkers for disease diagnosis. Changes to the concentration of metabolites will occur because of changes in the body in response to a disease [6–8]. We have developed a targeted liquid chromatography–tandem mass spectrometry (LC-MS/MS) platform for urine analysis to potentially differentiate people with respiratory diseases. The main advantage of urine over other biofluids for biomarker discovery is that it can be collected noninvasively, unlike serum or plasma [9–12]. Urine also represents an end-process biofluid impacted by multiple systems of the body, containing metabolites with a vast array of physiochemical properties [9, 13].

However, the use of urine in targeted LC-MS/MS analysis requires data normalization to account for variation in the urine concentration of study subjects [10–15]. Hydration affects the concentration of metabolites in urine and acts as a major source of potential variation [7, 10–12, 14]. As a result, several methods are used to account for the urine concentration or urine dilution of patients in urine metabolomics [14–16]. The most widely adopted data normalization strategy is the use of urine creatinine (CRN), which reflects urine concentration [10, 12, 14–18]. Other frequently used methods include measuring urine osmolarity and specific gravity [14–16]. There is no consensus on what normalization method is ideal in children or which metabolites are clinically relevant [19–22].

Normalizing urine concentration with CRN adds another level of challenge in children due to the variation in age and size. CRN excretion in urine is affected by height (HT) and muscle mass [18, 23]. In our lab, we became concerned that differences in CRN levels between children of different age groups/HTs was affecting our ability to differentiate children with asthma from healthy children across a range of age groups [18, 23]. There is no consensus on how to account for lean muscle mass differences in urine normalization. CRN relative to HT could be used to mediate the problem.

In addition, a newer approach for data normalization is total metabolome (TM) [14, 15, 24–26]. TM measures a subgroup of metabolites that contain amino and phenol functional groups as a representative subset of the overall (total) metabolome [14, 15]. TM normalization is not dependent on one metabolite measurement, like CRN, and is less affected by HT [14, 15, 25]. TM data normalization may provide an effective alternative to CRN or other normalization techniques among children.

The goal of this study is to compare the utility of both CRT/HT and TM data normalization approaches to CRN-only normalization in LC-MS/MS urine diagnostics in children across a wide range of ages and HTs. We hypothesized that using CRT/HT and TM would result in improved separation of children’s urine data based on disease status across samples of varying ages compared to conventional CRN data normalization strategies.

## 2. Materials and Methods

### 2.1. Materials

All analytical standards used for metabolite stocks and chemicals were obtained from Sigma (Oakville, ON, Canada) unless otherwise specified. CRN was purchased from Spectrum Laboratories Inc. (Rancho Dominguez, CA, United States); sarcosine from Santa Cruz Biotechnology (Santa Cruz, CA, United States); 1-methylnicotinaminde-d3, choline-d9 chloride, creatinine-d3 monohydrate, glucose-d2, guanidine acetic acid-d2, and uracil-d2 from Isotope Inc. (Pointe-Claire, QC, Canada); pyruvic acid-^13^C3 sodium salt from Omicron Biochemical Inc. (South Bend, IN, United States); and B-hydroxyisovaleric acid from Alfa Aesar (Ward Hill, MA, United States). Dansyl chloride (DnsCl) and ^13^C-dimethylamino phenacyl bromide (DmPA) were synthesized in-house according to previously published methods [12, 27, 28]. ^12^C-DmPA was purchased from Combi-Blocks Inc. (San Diego, CA, United States). Formic acid was purchased from Fisher (Ottawa, ON, Canada).

### 2.2. Patient Characteristics, Urine Collection, and Ethics Statement

All children were recruited after having received consent from their parents, as approved by the Biomedical Research Ethics Boards at the University of Saskatchewan (Bio13-89). Ethical approval for the CHILD Cohort Study, including the use of biological samples and the CHILD database (CHILDdb), was also obtained from the local research ethics board of each study site: the University of British Columbia, the University of Alberta, the University of Manitoba, the Hospital for Sick Children, and McMaster University. The CHILD Cohort Study emphasizes participant and public involvement in all stages of its research. Research questions are informed and generated based on relevance to participants and their families to study those affected by childhood health conditions. Efforts are made to ensure participants’ involvement is meaningful, comfortable, and aligned with their preferences. Study results are shared with participants through various channels, including newsletters and online platforms; academic and public health publications also communicate findings. We acknowledge and thank all participant advisors and study participants for their valuable contributions. Urine samples were collected midstream from children 5 years of age and older and via cotton swabs from diapers in 3-year-old children. All urine samples were collected and stored on ice before being placed at **–**80 °C within an hour. Samples were stored at –80 °C until analysis. Stability of metabolites and freeze-thaw cycles for urine samples was followed as previously published [10, 12, 17].

A summary of sample sizes of different ages and diagnosis is provided in Table 1. One cohort of children of an older age range (5–13 years) with or without an asthma diagnosis was recruited from the outpatient clinic, Department of Pediatrics, University of Saskatchewan. The healthy older children were recruited from the orthopedics follow-up clinic, having denied any previous history of allergy or asthma (n = 18, aged 5–10 years). The asthmatic older children (n = 29, aged 5–13) were recruited in an asthma clinic based on their history of asthma, medications, evidence of bronchodilator response on spirometry, and skin-test reaction to at least one aeroallergen. The second cohort of children was collected from the CHILD (Canadian Healthy Infant Longitudinal Development) study [29]. Clinical data and urine samples were collected in the same children at the age of 3 years (n = 100) and later at 5 years old (n = 99). At the age of 5, children were classified as healthy or asthmatic based on a patient history of recurrent wheezing, emergency room visits for asthma, use of asthma medications, and evidence of atopy on skin tests to aeroallergens. All healthy children had no such history according to the questionnaires.

**Table 1.** Sample sizes of all children used for comparisons of creatinine (CRN), creatinine-to-height (CRN/HT), and total metabolome (TM) based on health category and age.

Age category	Number asthmatic children for CRN	Number healthy children for CRN	Total children for CRN	Number asthmatic children for TM	Number healthy children for TM	Total children for TM
Older (5–13 y)	29	18	47	29	18	47
5 years of age	49	50	99	13	13	26
3 years of age	50	50	100	18	12	30

Unfortunately, TM analysis was not possible for all the CHILD samples. By the time we decided to study TM, we did not have enough urine volume for many 3- and 5-year-old children. Thus, the total cohort size for metabolomic analysis decreased for the CHILD samples (Table 2). There was no discernable relationship between age or health status for these samples compared to the total cohort. There was limited age overlap, and only five children from the older cohort were 5 years old. This limited overlap should mitigate any potential bias. These samples were also separated as they were collected at a different hospital from the 5-year-old cohort.

**Table 2.** Samples sizes of the model building and blinded test sets as separated based on their health category and age.

	Older healthy model	Older asthma model	Older healthy blinded	5-year-old asthma blinded	3-year-old healthy blinded	3-year-old asthma blinded
Older (5–13 years) (N =)	4	29	14	0	0	0
5 years of age (N =)	13	0	0	13	0	0
3 years of age (N =)	0	0	0	0	18	12

### 2.3. LC-MS Analysis

LC-MS/MS analysis was performed on an Agilent 1290 ultra performance liquid chromatography (UPLC) coupled to a triple quadrupole-linear ion trap MS instrument, namely SCIEX 6500 QTRAP®, as per published methods targeting 41 metabolites [10, 12, 17]. In short, three unique validated quantitative methods were used to determine the concentration of 41 metabolites using LC-MS/MS (Table 2). A hydrophilic interaction liquid chromatography–mass spectrometry (HILIC-MS) method was used to separate and quantify seven polar metabolites [15]. A C-18 UPLC column was used to quantify 17 organic acid metabolites, derivatized with 4-DmPA [16]. A further 17 metabolites containing amine and phenol groups were derivatized with DnsCl for quantification using a UPLC C-18 column [14]. A summary of quantified metabolites using each method is outlined in Table 3. Quantified metabolites identified as potential diagnostic biomarkers are based on previous research [30, 31].

**Table 3.** List of metabolites quantified using LC-MS methods.

UPLC-MS (DnsCl)	UPLC-MS (DmPA)	HILIC-MS
1-Methylhistamine	Betaine	1-Methylnicotinaminde
Alanine	Trigonelline	Choline
Arginine	O-Acetyl carnitine	Guanidine acetic acid
Asparagine	Glycolic acid	Glucose
Ethanolamine	Pyroglutamic acid	Creatine
Glycine	Lactic acid	Pyruvic acid
Glutamine	3-Hydroxybutryic acid	Uracil
Histidine	Pantothenic acid	
Isoleucine	2-Hydroxyisobutyric acid	
Lysine	3-Hydroxyisovaleric acid	
Sarcosine	2-Oxoglutaric acid	
Serine	3-Hydroxy-3-methylglutaric acid	
Threonine	Succinic acid	
Tyrosine	Fumaric acid	
Tryptophan	3-Methyladipic acid	
Taurine	Cis/trans aconitic acid	
Valine		

41 metabolites were quantified using three LC-MS/MS methods [10–12]. Amine- and phenol-containing metabolites derivatized with dansyl chloride (DnsCl) are outlined in column 1. Organic acid metabolites derivatized with dimethylamino phenacyl bromide (DmPA) for quantification are outlined in column 2, and polar metabolites quantified using a HILIC-MS without derivatization are outlined in column 3.

### 2.4. Data Normalization

#### 2.4.1. CRN-based normalization

CRN was measured using a QuantiChrom™ Creatinine Assay Kit (QC, Canada) and a BioTek plate reader (BioAssay Systems, Hayward, CA, United States) at 510 nm following the manufacturer’s protocol. The assay is based on Jaffe’s reaction, detected at 510 nm [32]. Measured CRN values were used as a normalization factor for each individual LC-MS/MS biomarker. A second normalization method incorporated both CRN concentration and child HT, with HT included to account for variation in growth. HT was measured in the clinic at the time of urine collection. A CRN-to-HT (CRN/HT) ratio was used to normalize each individual LC-MS/MS biomarker concentration, reducing variability in CRN excretion among children. All CRN and LC-MS/MS metabolite values were within the acceptable analytical range or diluted to within the range for all samples.

#### 2.4.2. Total metabolome

The TM was quantified using LC with ultraviolet (UV) detection on an Agilent 1200 HPLC-UV with a Kintex C-18 column (5 µm × 100, 2.1 mm). The procedure for measuring the TM was adapted from Wu and Li 2012 with the following minor modifications [15]. A total of 24 amine- and phenol-containing metabolites were used to create the standard (Supplementary Table 1). Seven additional metabolites were added to better replicate the diversity of the metabolome in urine for a total of 24 metabolites in the standard curve created based on previous research [15]. The standard curve was created in 50% acetonitrile (ACN) solvent and derivatized using DnsCl as per the method outlined by Khamis et al. [10]. This standard curve was used to determine the concentration of TM in each urine sample.

Urine samples were diluted 1:3 with ACN and centrifuged at 14,000 rotations per minute for 10 min. The supernatant was then used for derivatization as described [10]. To account for the matrix effects of salts in urine, the solvent standard curve was matched to a threefold diluted urine standard curve [33]. The measurement of underivatized urine signal for each sample was subtracted to remove any confounding metabolite chromophore contribution to the value of the TM, measured upon derivatization. The total signal from the derivatized metabolites following the removal of the underivatized signal contribution was used to represent the TM value for data normalization. This value was used to normalize each individual LC-MS/MS biomarker concentration. The selected samples contained TM values within the limits of sensitivity (0.89–5.32 mM).

### 2.5. Statistical Analysis

HT, CRN, CRN/HT, and TM values were compared between each of the three age groups: 3-year-olds, 5-year-olds, and older children (5–13 years) using the nonparametric Kruskal-Wallis test with multiple comparisons. LC-MS/MS metabolite concentrations were normalized post-acquisition using three approaches: (1) to CRN alone, (2) to CRN/HT, and (3) to TM. To distinguish metabolite profiles between healthy and asthmatic children, the normalized values were analyzed by orthogonal partial least squares–discriminant analysis (OPLS-DA) using SIMCA® software (SIMCA-P 18, Umetrics, Sweden). This process identifies metabolites whose concentrations differ significantly between groups of patients, creating predictive models with correlation coefficients (R^2^) and prediction properties (Q^2^). The OPLS-DA generates a prediction score (0–1) for each sample, where scores less than 0.5 indicate classification as healthy rather than asthmatic (>0.5). Metabolites with consistently greater differences in concentration between groups are displayed as a variables of importance plot (VIP). To assess the predictive validity of the metabolite models, urine samples not included in the training model were analyzed blindly without a diagnosis. The test set of samples contained n =12 for healthy 3-year-olds and n = 18 for asthmatic 3-year-olds, n = 14 for healthy 5–13-year-olds, and n = 13 for asthmatic 5-year-olds. Samples were selected for training sets based on the confidence of their clinical diagnosis. Each urine sample was scored relative to the model. We previously reported using this technique in an animal model of asthma and children with asthma [30, 31].

## 3. Results

### 3.1. Comparison of Normalization Strategies Across Age Groups

CRN concentrations were compared between three age cohorts: 3-year-olds, 5-year-olds, and older children (5–13 years). As expected, taller older children had higher CRN levels than shorter, younger children, and the discrepancy was specifically evident in the older group (5–13 years), where CRN was highest (Figure 1A and 1B). To address this, we normalized CRN/HT. CRN/HT did diminish the differences between ages, but in the extremes comparing 3-year-olds versus older groups there remained a small, but statistically significant, difference (Figure 1C). In contrast, TM showed no significant differences across ages (Figure 1D).

**Figure 1.**
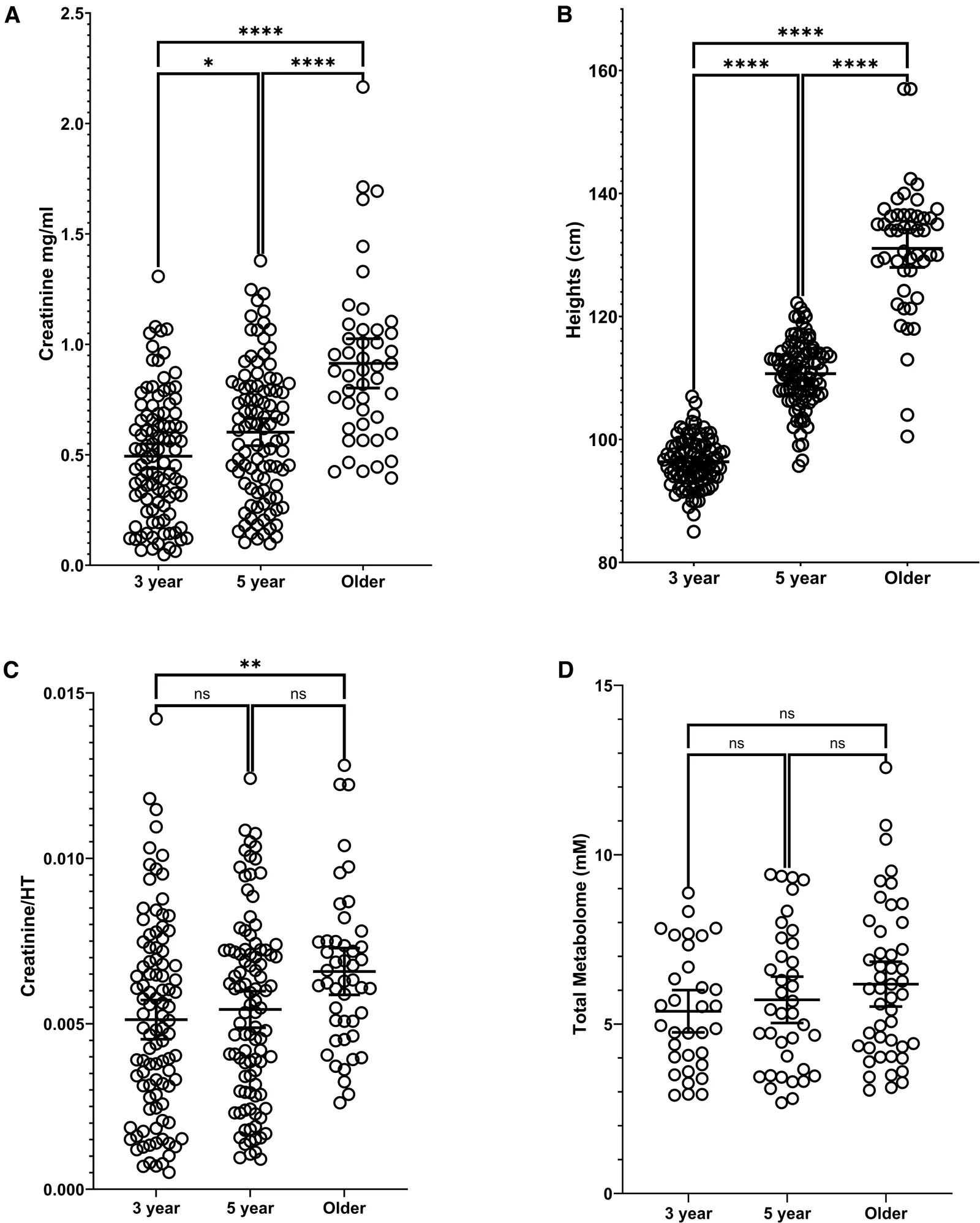
Comparison of height (HT) and normalization values of children across age cohorts. The Creatinine (A) and HT (B) values are shown for the three age groups: 3-year-olds (n = 100), 5-year-olds (n = 99), and 5–13-year-old children (older, n = 47). Errors bars are the standard error mean. Values increase across age cohorts. CRN/HT (C) shows a significant difference only between the 3-year-old and older cohort. Sample size for each age group: 3-year-olds (n = 100), 5-year-olds (n = 99), and 5–13-year-olds (older, n = 47). Based on available urine samples, Total Metabolome (D) shows no significant difference across any age category; 3-year-olds (n = 30), 5-year-olds (n = 26), and older (n = 47). * Indicates p ≤ 0.05; ** Indicates p ≤ 0.01; **** Indicates p ≤ 0.0001.

### 3.2. Studying Diagnostic Potential for Each Normalization Strategy

We then used OPLS-DA to examine potential diagnostic models between each disease state based on differences in metabolite values normalized to CRN, CRN/HT, or TM. The older children in our study had a confirmed asthma diagnosis based on lung function, which was missing in the younger groups (ages 3 and 5). As such the older group was considered the gold standard for asthma diagnosis. We used the healthy 5-year-olds (n = 13) and some healthy older children (n = 4) and the asthmatic older group (n = 29) to create a potential diagnostic model. Each of the OPLS-DA models were then tested for potential diagnostic accuracy using the remaining children (n = 14 older healthy, n = 13 5-year-old asthma, n = 18 3-year-old healthy, n = 12 3-year-old asthma). Urine data from the remaining groups of children across the range of ages and clinical diagnoses were presented to the model blinded without diagnostic classification. The SIMCA® model scored each sample as asthmatic or healthy. Healthy children were expected to have a predictive score less than 0.5, while asthma would be above 0.5. Predicting healthy children is the goal of any diagnostic. Metabolites with low importance (VIP < 0.7) or those contributing to misclassification of healthy were removed from the model, so long as the R^2^Q^2^ remained strong.

The CRN model was weakest in model strength (R^2^ = 0.70, Q^2^ = 0.49), and it had the weakest accuracy. This was especially evident in the 3-year-old children. For example, the percent accuracy for the CRN model in correctly identifying older healthy children was only 11 out of 14 (79%) and worse in the 3-year-old healthy group with only 5 out of 12 (42%) correctly scored. The accuracy data can be visualized in Figure 2. In contrast, CRN/HT and TM had stronger model strength (R^2^ = 0.87, Q^2^ = 0.64, and R^2^ = 0.89, Q^2^ = 0.64, respectively) and both had greater accuracy in predicting healthy children. CRN/HT and TM both predicted 12 out of 14 (86%) correctly in the older healthy kids. In the 3-year-olds, CRN/HT correctly predicted 11 out of 12 (92%), and TM produced similar data with 10 out of 12 (83%). Overall, both alternatives to traditional CRN showed an improvement in preventing misdiagnosis compared to CRN alone.

**Figure 2.**
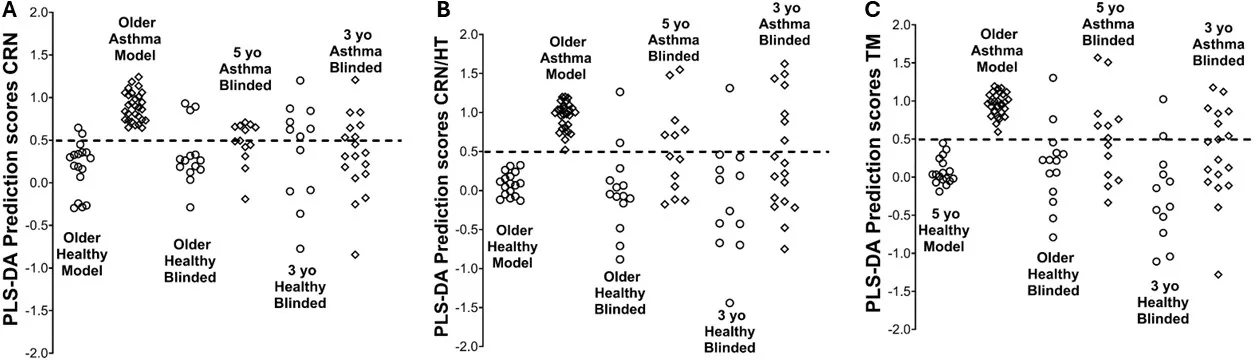
OPLS-DA Prediction plots created for creatinine (CRN), creatine-to-height (CRN/HT), and total metabolome (TM). Values below 0.5 are designated healthy, and values above 0.5 indicate asthmatic children. Samples are separated into those used for model creation and blinded older (5–13-year-old) and younger (3-year-old) children. Panel A shows the CRN-normalized model. Panel B shows the CRN/HT normalized model. Panel C shows the TM-normalized model.

Unfortunately, none of the modeling strategies could accurately diagnose asthma at any age. In 5-year-olds classified as having asthma, CRN only identified 6 out of 13 (46%) correctly, CRN/HT 6 out of 13 (46%), and TM 7 out of 13 (54%). Similarly, in the 3-year-olds, asthma was poorly identified, with CRN at 7 out of 18 (39%) correct, CRN/HT at 7 out of 18 (39%) correct, and TM at 8 out of 18 (44%) correct. Overall, CRN showed the least accurate predictive capacity of all three normalization techniques at any age when using an asthma model to compare normalization techniques. There are factors to consider regarding the poor accuracy for asthma diagnosis, which are outlined in the “Discussion.”

### 3.3. Comparison of Metabolites in Healthy Versus Asthma Using Various Normalization Approaches

The specific importance of the metabolites needed to create each model differed depending on the normalization technique (Table 4). The metabolites contributing to the model (VIP > 0.7) were similar but not the same for all strategies, as each model was individually refined to maximize diagnostic accuracy and compare the effect of normalization on the creation of a final model (Supplementary Figure 1). After each model was refined, the final metabolite profiles were compared. As seen in Supplementary Figure 1 and Supplementary Table 2, a total of 10 metabolites were shared between all three models (bolded metabolites). In fact, a total of 6 metabolites were shared between CRN alone and TM models (*), and 5 metabolites were shared between CRN/HT and TM (ǂ). Interestingly, only 1 metabolite was uniquely shared between both CRN-based methods (italicized). The number of metabolites used in each model also varied. CRN had a total of 26 metabolites required for model creation, and 18 and 24 metabolites were required for CRN/HT and TM, respectively (Table 4).

**Table 4.** Metabolites above threshold of significance for various normalization strategies.

Metabolites of significance normalized to CRN	Metabolites of significance normalized to CRN/HT	Metabolites of significance normalized to TM
**Choline**	**Uracil**	**Tyrosine**
**Uracil**	**Tyrosine**	**Histidine**
Pyruvate	Ethanolamine	**Tryptophan**
Betaine	**Tryptophan**	**Threonine**
Glucose*	**Choline**	Asparagine*
**Tyrosine**	**Threonine**	Arginine*
*3M-3-OH-glutarate*	**Valine**	Serineǂ
Sarcosine	**Histidine**	Lysine
**Tryptophan**	Serineǂ	**Glutamate**
Succinate	1M-Histamineǂ	**Valine**
**Histidine**	**Glutamate**	Isoleucine*
Trigonelline	*3M-3OH-glutarate*	1M-Histamineǂ
**Guanidine acetate**	2-OH-butyateǂ	2OH-butyrateǂ
Pyroglutarate	Succinate	Glycine*
**Threonine**	**Alanine**	**Alanine**
Lactate	Taurineǂ	**Uracil**
2-Oxoglutaric	Glycolic acidǂ	Taurineǂ
Asparagine*	**Guanidine acetate**	Pyroglutarate
**Glutamate**		Pantothenate*
**Alanine**		Glycolic acidǂ
Arginine*		Glucose*
Glycine*		Acetylcarnitine
3M-adipate		**Guanidine acetate**
Isoleucine*		**Choline**
Pantothenate*		
**Valine**		

Metabolites of significance from OPLS-DA analysis above the cutoff value (VIP > 0.7) for each normalization technique listed in order of importance. The left column shows the 26 metabolites normalized to CRN alone. The middle column shows the 18 metabolites normalized to CRN/HT. The right column shows the 24 metabolites normalized to the TM. Bolded metabolites denote metabolites shown between all three methods.

* Denotes metabolites shared between CRN and TM methods.

ǂ Denotes metabolites shared between CRN/HT and TM methods.

Italicized metabolites denote metabolites shared between CRN and CRN/HT methods.

## 4. Discussion

### 4.1. Effect of Age on Normalization Value

The normalization strategies were compared between age groups to compare the effect of HT/muscle mass. The significant variation seen between age groups when using urinary CRN alone to account for urine concentration indicated that CRN alone is less accurate compared to CRN/HT and TM (Figure 1). This variation could affect urinary LC-MS/MS data when we compare children with significant variation in HT/muscle mass. CRN/HT was chosen to account for variation in CRN across ages and showed less variation between age categories than CRN alone. CRN/HT showed no significant difference between 3- and 5-year-olds and the 5-year-old and 5–13-year-old (older) children (Figure 1). This suggested that dividing CRN to HT partially corrects for age-based variation. However, the variation seen when comparing the youngest and oldest cohorts indicates that CRN/HT is still affected by more significant size differences. TM showed no significant differences when compared between any age categories (Figure 1). This indicates that TM was the least affected by age or HT-related variation. We suggest that TM can be considered less affected by age and HT compared to traditional CRN methods. Both CRN/HT and TM showed improvement when compared to traditional CRN in the context of a pediatric asthma model.

### 4.2. Effect of Normalization on Diagnostic Accuracy

The goal of any test is to first avoid misclassification as disease when the subject is healthy. The effect of normalization on model accuracy was compared (Figure 2). Each model was created using the older children with asthma, where the diagnosis was best confirmed. Diagnosing children as asthmatic is more difficult in younger children (<6 years) as they are not able to perform lung function tests. The samples selected for the healthy model training set were chosen to include age variation. As such we added a small number of children from the older cohort (n = 4) to the training set of healthy 5-year-olds. This allowed for the test set to incorporate a wider age range for healthy children. The model created with each normalization technique varied in its capacity to correctly predict across different age groups, indicating that normalization affects model creation. CRN had the least accurate prediction compared to CRN/HT or TM, indicating that CRN is a poor normalization choice for pediatric patients across wider age groups. CRN/HT and TM had improved capacity to avoid the misclassification of healthy individuals in both the younger and older groups. TM showed mild improvement in diagnosing asthma compared to CRN/HT, but all the models struggled to predict an asthma diagnosis in younger children. This may be due to the difficulty of diagnosing asthma in younger children because of limited lung function data. It is possible that the 5-year-olds and certainly 3-year-olds did not have asthma and were misdiagnosed as asthmatic during the recruitment. Classifying the 5-year-old as having asthma was based on the physician diagnosis of asthma and evidence of atopy on a skin prick test. This diagnosis of asthma at that age is not as strong as in the older population with lung function data. Nonetheless, the diagnosis at this age was considered reasonable based on a history of recurrent wheezing, use of medications for asthma, and positive skin prick tests for atopy. The lack of spirometry could also contribute to the weakness of the models to diagnose asthmatic 3-year-olds. It is also possible that the physiology of very young children (i.e., 3-year-olds) and older children extends beyond the presence or absence of asthma, altering metabolite composition and contributing to variation in the metabolome, which in turn may reduce diagnostic accuracy. The main objective of this study was to determine if TM or CRN/HT would improve the normalization of urine concentration. The second objective was to determine if this improved the diagnosis of asthma in pediatric patients. Repetition of this approach with the 5-year-old samples at an older age with spirometry results confirming the asthma diagnosis (positive controls) is the next step and will be conducted in the future.

### 4.3. Effect of Normalization on Metabolite Profile in Model Refinement

In addition to comparing the accuracy of each normalization technique’s model, the metabolites used to create the models were analyzed. The models varied in both the number of metabolites and the metabolites required for creating the best refined model (Table 4). Interestingly, CRN/HT shared more metabolites in common with TM than CRN alone. This indicates that both TM and CRN/HT provide improved methods for correcting urine concentration. A total of 10 metabolites were found to be shared between all three normalization models (Table 4). This substantial overlap in metabolites across models supports the possibility of defining a diagnostic profile that distinguishes asthmatic from healthy children. Models created for each normalization technique using only the 10 shared metabolites are shown in Supplementary Figure 2. These models were still unable to fully distinguish healthy from asthmatic children with complete accuracy. However, the variation in the strength of certain metabolites across models, along with the presence of unique metabolites introduced by each normalization method, supports the conclusion that diagnostic performance is influenced by the choice of normalization. A summary of metabolites removed from each model is provided in the supplementary materials (Supplementary Table 2). A total of five metabolites were removed from all models.

### 4.4. Other Factors to Consider

The models were refined to maximize diagnostic capability, but the presence of consistently poor diagnostic accuracy of 3-year-olds across the various models could be explained by other factors. Urine samples from 3-year-olds were collected from diapers, which may not provide the level of quality required for diagnostic purposes. The quality of the data could have been affected by potential contamination and delayed freezing during collection. Samples were stored for 5 years prior to TM analysis.

## 5. Conclusion

The choice of the normalization strategy is an important consideration when investigating potential urine diagnostics. This is particularly important when comparing pediatric patient groups with significant differences in HT due to age differences. This research found significant differences in the metabolomic models depending on the normalization method applied to the same set of LC-MS/MS urine data. Age and HT influenced CRN-only normalization in children of varying sizes and were a potential confounder in model development. This effect was mitigated by normalizing to CRN/HT or TM. CRN/HT and TM lead to similarly improved healthy diagnosis despite age variation compared to CRN alone. However, the TM method is hampered somewhat by longer processing times and more extensive sample preparation compared to CRN/HT. The observed changes in model separation as a result of normalization highlight the need for careful consideration when choosing a normalization strategy in pediatric metabolomics. While CRN is the standard method for accounting for the urine concentration of a study subject, it may be less appropriate in populations with wide variability in body size (i.e., muscle mass), such as children. Based on our findings, CRN/HT or TM are recommended for normalization in pediatric metabolomic research.

## Supplementary Material

Supplementary Material

## Data Availability

Data described in the manuscript are available via registration to the CHILD database at https://childstudy.ca/childdb/ and the submission of a formal request. More information about data access for the CHILD Cohort Study can be found at https://childstudy.ca/for-researchers/data-access/. Researchers interested in accessing CHILD Cohort Study data for their research should contact child@mcmaster.ca.

## Abbreviations

ACN: Acetonitrile
CRN: Creatinine
CRN/HT: Creatinine to height
DmPA: Dimethylamino phenacyl bromide
DnsCl: Dansyl chloride
HILIC-MS: Hydrophilic interaction liquid chromatography–mass spectrometry
HT: Height
LC-MS: Liquid chromatography–mass spectrometry
LC-MS/MS: Liquid chromatography–tandem mass spectrometry
OPLS-DA: Orthogonal partial least squares–discriminant analysis
TM: Total metabolome
UPLC: Ultra performance liquid chromatography
UV: Ultraviolet
VIP: Variables of importance plot

## Supplementary Material

Supplemental Material Available at https://doi.org/10.21627/3p72dt97.
