# Supplementary material for: Comparison of Three Urine Normalization Strategies for Mass Spectrometric Analysis in a Pediatric Asthma Model
Source: J Mass Spectrom Adv Clin Lab. 2026 Sep 15;39(1):3p72dt97. doi: 10.21627/3p72dt97 (PMC13750509; doi:10.21627/3p72dt97)
Supplement: Supplementary file 1 [file jmsacl-39-1-3p72dt97-s001.docx]

Supplementary Materials

Comparison of Three Urine Normalization Strategies for Mass Spectrometric Analysis in a Pediatric Asthma Model

Emma Finch^1^, Maryam Alyari^1,2^, Mays Al-Dulaymi^1^, Anas El-Aneed^2^ and Darryl J Adamko*^1^

^1^Department of Pediatrics, College of Medicine, ^2^College of Nutrition & Pharmacology, University of Saskatchewan, Saskatoon, Saskatchewan, Canada.

**Metabolites Used in Total Metabolome Standard Curve Creation**

Sarcosine

1-Methyl Histamine

Isoleucine

Valine

Histidine

Threonine

Tryptophan

Alanine

Glutamine

Tyrosine

Serine

Arginine

Glycine

Ethanolamine

Taurine

Asparagine

Lysine

Cysteine

Phenylalanine

Methionine

Leucine

Aspartic Acid

Glutamic Acid

Proline

**Supplementary Table 1.** Total Metabolome Standard Curve Metabolites. A list of the 24 metabolites involved in the standard curve creation for the quantification of the total metabolome.

 **Supplementary Figure 1.** Variables of Importance Plots (VIP) Normalizing Data with Creatinine (A), Creatinine to Height (B) and Total Metabolome (C). Models were refined to include metabolites with a VIP score >0.7.

**Supplementary Figure 2 OPLS-DA Prediction Plots Created for Creatinine, Creatinine/ Height and Total Metabolome Using Shared Metabolites**. Values below 0.5 are designated healthy and above 0.5 asthmatic children. Samples are separated into those used for model creation and blinded older (5-14-year-old) and younger (3-year-old) children. A shows creatinine normalized model with only the metabolites common between all optimized models. B shows creatinine to height normalized model with only the metabolites common between all optimized models. C shows total metabolome normalized model with only the metabolites common between all optimized models.

| **CRN Alone Removed Metabolites** | **CRN/Ht Removed Metabolites** | **TM Removed Metabolites** |
| --- | --- | --- |
| *Acetylcarnitine* | *Acetylcarnitine* | **Cis/Trans Aconitate** |
| **Cis/Trans Aconitate** | **Cis/Trans Aconitate** | 3-Methyl Adipate ǂ |
| 2-Hydroxy-Butyrate | 3-Methyl Adipate ǂ | Betaine ǂ |
| **3-Hydroxy Butyrate** | Arginine | **3-Hydroxy Butyrate** |
| **Creatine** | Asparagine | **Creatine** |
| Ethanolamine* | Betaine ǂ | Ethanolamine* |
| Glycolic Acid | **3-Hydroxy Butyrate** | 3-Methyl-3-Hydroxy Glutarate |
| 1-Methyl Histamine | **Creatine** | **Isovalerate** |
| **Isovalerate** | Glucose | Lactate ǂ |
| *Lysine* | Glycine | **1-Methyl Nicotinamide** |
| **1-Methyl Nicotinamide** | Isoleucine | 2-Oxoglutarate ǂ |
| Serine | **Isovalerate** | Pyruvate ǂ |
| Taurine | Lactate ǂ | Sarcosine ǂ |
|  | *Lysine* | Succinate |
|  | **1-Methyl Nicotinamide** | Trigonelline ǂ |
|  | 2-Oxoglutarate ǂ |  |
|  | Pantothenate |  |
|  | Pyroglutamate |  |
|  | Pyruvate ǂ |  |
|  | Sarcosine ǂ |  |
|  | Trigonelline ǂ |  |

**Supplementary Table 2.** **Removed Metabolites Below Threshold of Significance for Various Normalization Strategies**. Metabolites were removed from oPLS-DA analysis if they fell below the cut off value (VIP >0.7) for each normalization technique. Left column shows the total of 13 removed metabolites normalized to creatinine alone. Middle column shows the total of 21 removed metabolites normalized to creatinine to height. Right column shows the 15 removed metabolites normalized to total metabolome. Bolded metabolites denote metabolites removed in all three methods. * denotes metabolites removed in both CRN and TM methods. ǂ denotes metabolites removed in both CRN/HT and TM methods. Italicized metabolites denote metabolites removed in both CRN and CRN/HT methods.
